# A Single Nucleotide Polymorphism (rs4236480) in *TRPV5* Calcium Channel Gene Is Associated with Stone Multiplicity in Calcium Nephrolithiasis Patients

**DOI:** 10.1155/2015/375427

**Published:** 2015-05-18

**Authors:** Anas Khaleel, Mei-Shin Wu, Henry Sung-Ching Wong, Yu-Wen Hsu, Yii-Her Chou, Hsiang-Yin Chen

**Affiliations:** ^1^Department of Clinical Pharmacy, School of Pharmacy, Taipei Medical University, Taipei 11031, Taiwan; ^2^Master's Program for Clinical Pharmacogenomics and Pharmacoproteomics, School of Pharmacy, Taipei Medical University, Taipei 11031, Taiwan; ^3^Department of Urology, College of Medicine, Kaohsiung Medical University, Kaohsiung 80756, Taiwan; ^4^Department of Urology, Kaohsiung Medical University Hospital, Kaohsiung 80756, Taiwan; ^5^Department of Pharmacy, Wan Fang Hospital, Taipei Medical University, Taipei 11696, Taiwan

## Abstract

Nephrolithiasis is characterized by calcification of stones in the kidneys from an unknown cause. Animal models demonstrated the functional roles of the transient receptor potential vanilloid member 5 (*TRPV5*) gene in calcium renal reabsorption and hypercalciuria. Therefore, *TRPV5* was suggested to be involved in calcium homeostasis. However, whether genetic polymorphisms of *TRPV5* are associated with kidney stone multiplicity or recurrence is unclear. In this study, 365 Taiwanese kidney-stone patients were recruited. Both biochemical data and DNA samples were collected. Genotyping was performed by a TaqMan allelic discrimination assay. We found that a *TRPV5* polymorphism (rs4236480) was observed to be associated with stone multiplicity of calcium nephrolithiasis, as the risk of stone multiplicity was higher in patients with the TT+CT genotype than in patients with the CC genotype (*p* = 0.0271). In summary, despite the complexity of nephrolithiasis and the potential association of numerous calcium homeostatic absorption/reabsorption factors, *TRPV5* plays an important role in the pathogenesis of calcium nephrolithiasis.

## 1. Introduction

Nephrolithiasis is a disease caused by calcifications that form stones in the kidney. The major component of these stones is calcium. Nephrolithiasis is a globally common disorder, and the prevalence and incidence of nephrolithiasis have been dramatically increasing worldwide. This phenomenon has placed heavy burdens on national economics and healthcare systems [1, 2]. Many factors were suggested to be risk factors for nephrolithiasis, such as a family's health history, environmental exposure, for example, to steel manufacturing [3], dietary intake [4–7], a low fluid intake [8], and hypercalciuria [9–12]. Genetic polymorphisms were also reported to be risk factors causing kidney-stone formation and elevating nephrolithiasis recurrence. For example, several genetic variations of the calcium-sensing receptor (*CASR*) gene were reported to be associated with an increase in recurrent calcium kidney-stone formation [13, 14]. Genetic polymorphisms of* Orai1*, a major subunit of the store-operated calcium channel, were also reported to be associated with nephrolithiasis recurrence [15]. In addition, levels of the vitamin D receptor (*VDR*) and its genetic polymorphisms were also indicated to be associated with idiopathic hypercalciuria (IH) [16, 17].

The transient receptor potential cation channel, subfamily V, member 5 (*TRPV5*) is considered the gatekeeper channel for activating calcium transport and reabsorption [18, 19].* TRPV5* channel was found to be associated with urinary calcium (re)absorption in protein expression and channel voltage experiments [20, 21]. This channel is expressed in the epithelia of renal connecting tubules and distal convoluted tubules, which are major parts of the kidneys involved in calcium homeostasis [22–24]. A previous study indicated that a* TRPV5* genetic polymorphism has a conservative calcium effect in African subjects, and an animal model showed that genetic knockdown in mice produced serious hypercalciuria, which is a major risk factor for kidney-stone formation [18, 19]. Despite the study results of Renkema et al. not supporting a functional role for* TRPV5* in the pathogenesis of renal hypercalciuria, they still suggested that the* TRPV5* gene should not be excluded as a potential risk factor for hypercalciuria [25].

The goal of this study was to investigate associations of the single-nucleotide polymorphism (SNP), rs4236480, of* TRPV5* with the stone number (multiplicity) and recurrence rate in a Taiwanese population.

## 2. Material and Methods

### 2.1. Patient Recruitment and Study Methods

There were 365 renal-stone patients who fulfilled the diagnostic criteria for nephrolithiasis and were enrolled in this study. Sample collection was approved by the Institutional Review Board of Kaohsiung Medical University Hospital (KMUH) and patients were recruited after signing informed consent. Subjects were excluded if they had a history of renal tubular acidosis, primary and secondary hyperparathyroidism, renal failure, gout, chronic diarrhea, or cancer. We excluded patients with other stone compositions and only recruited patients with idiopathic calcium nephrolithiasis.

Each patient's radiographic and echographic examinations of renal stones were retrieved along with clinical laboratory data, such as age, sex, and family history of nephrolithiasis. Renal-stone samples were obtained by either spontaneous stone passage or surgical intervention. Moreover, in stone number examination, kidney-stone patients with a single stone were allocated to single stone group, while kidney-stone patients having stone numbers exceeding one were allocated to multiple stone group.

The past medical history for renal-stone was retrospectively traced back as far as the patient's medical record was available. Patients who had two or more symptomatic episodes within at least a 6-month interval were classified to the recurrent group; those with only one episode were allocated to the nonrecurrent group. Due to the retrospective nature of the study, some subjects did not have clear past medical history documented in their medical records. Those patients who did not have clear documentations to classify them into either single or recurrent groups were excluded from the analysis for recurrence.

### 2.2. DNA Extraction

DNA was derived from whole-blood samples which were collected from patients. Blood cells were first treated with 0.5% sodium dodecylsulfate lysis buffer and then with protease K (1 mg/mL) for 4 h at 60°C to digest nuclear proteins. A Gentra (QIAGEN, Valencia, CA) extraction kit and 70% alcohol precipitation were used for DNA extraction by a method described in the previous study [26].

### 2.3. Genotyping for* TRPV5* Polymorphisms

The nonsynonymous SNP, rs4236480, of* TRPV5* was selected as the genotyping target. The minor allele frequency of this SNP is 10% among a Han Chinese population from Beijing, China, based on the HapMap project database (http://hapmap.ncbi.nlm.nih.gov/) [27]. Functionally this SNP represents a nonsynonymous polymorphism in intracellular N- and C-tails [25]. Genotyping was performed using the TaqMan allelic discrimination assay (Applied Biosystems, Foster City, CA). In short, a polymerase chain reaction (PCR) using a 96-well microplate with an ABI 9700 thermal cycler (Applied Biosystems) was performed for the purpose of target sequence enlargement. After the PCR, the fluorescence was measured and analyzed using system SDS software version 1.2.3 (Applied Biosystems). We followed the protocol which was previously reported [26].

### 2.4. Statistical Analysis

Statistical software R (version 3.0.3) and R package Bioconductor [28, 29] were used for data cleaning and statistical analyses. Genotyping and allelic frequencies between phenotypes were statistically analyzed by the likelihood ratio test. Each *p* value was adjusted for age and sex. A *p* value of <0.05 was considered statistically significant.

## 3. Results

### 3.1. Demographic and Clinical Features of Stone-Bearing Patients

**Figure 1:**
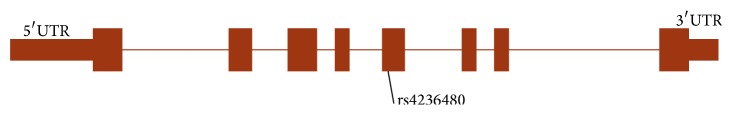
Graphical overview of the genotyped human* TRPV5* gene.

We recruited 365 kidney-stone patients for this study. The mean age of patients was 54.3 ± 12.0 (standard deviation) years, and 68.5% of patients were male (Table 1). As shown in the HapMap project database, rs4236480 of* TRPV5* has a 10% minor allelic frequency among a Han Chinese population from Beijing, China [27]. A graphical outline of the rs4236480 location is shown in Figure 1.

### 3.2. The* TRPV5* Genetic Polymorphism (rs4236480) Is Significantly Associated with Stone Multiplicity in Calcium Nephrolithiasis Patients

In order to investigate the association between stone multiplicity and genotypes, we further examined whether the* TRPV5* SNP rs4236480 genotypes were associated with the stone number of calcium nephrolithiasis patients. The T allele of the rs4236480 polymorphism is a risk allele that appeared to have a dominant genetic effect upon the C allele (Table 2). The risk for stone multiplicity was higher in patients who carried at least one minor allele than in the wild-type group, and a significant association between rs4236480 and kidney-stone multiplicity was observed (genotype model, *p* = 0.0372; dominant model, *p* = 0.0271; log additive model, *p* = 0.0167) (Table 2).

### 3.3. Lack of an Association of the* TRPV5* Genetic Polymorphism (rs4236480) with the Risk of Stone Recurrence in Patients with Calcium Nephrolithiasis

We suspected that SNP genotypes (of rs4236480) were associated with stone recurrence in calcium nephrolithiasis patients. However, the association of genotypes with stone recurrence of calcium nephrolithiasis did not reach statistical significance (Table 3).

### 3.4. No Association between the rs4236480 Polymorphism of* TRPV5* and Biochemical Data of Nephrolithiasis Patients

To better understand the interrelation between rs4236480 and clinical manifestation risk factors, biochemical data of serum/urine levels of calcium, uric acid, and creatinine were analyzed using urine samples obtained from patients on the spot and with creatinine correction. No significance was found among these clinical parameters (Table 4), and a subgroup analysis also produced no significant results (Table 5).

## 4. Discussion

We recruited 365 nephrolithiasis patients to conduct an association study between the* TRPV5* genetic polymorphism, rs4236480, and stone multiplicity/recurrence in calcium nephrolithiasis patients. We found a significant association between rs4236480 and stone multiplicity. To our knowledge, this is the first study to report a significant association of the* TRPV5* genetic polymorphism, rs4236480, with the types of kidney-stone multiplicity in Taiwanese calcium nephrolithiasis patients.

Genetic and environmental risk factors influence body levels of calcium ions considerably and thus contribute to calcium nephrolithiasis [30, 31]. Calcium is the predominant constituent of kidney-stone precipitates, as nearly 80% of stones are made of calcium, either mostly calcium oxalate or less frequently calcium phosphate [32–34]. High levels of urinary calcium are predominantly urine chemical aberrations in recurrent kidney-stone formers. Subsequently, hypercalciuria is considered to be the main risk factor for renal-stone formation. A family history of nephrolithiasis increases an individual's predisposition for a higher risk of kidney-stone formation [35, 36], which substantially implies a susceptibility to genetic causes. Polymorphisms in multiple genes have been recently reported to be associated with the renal stone formation, that include calcium-sensing receptor (*CASR*), vitamin D receptor (*VDR*), and osteopontin (*OPN*), inositol 1,4,5-trisphosphate (IP3) 3-kinase C (*ITPKC*), and* ORAI1* and claudin 14 (*CLDN14*) genes [15, 26, 37].

*TRPV5* is expressed in renal tubules and is regarded as a gatekeeper for calcium ion reabsorption [38, 39].* TRPV5* knockdown mice exhibited severe hypercalciuria [18]. This gene downregulates urinary reabsorption of calcium and exaggerates urinary excretion of calcium in genetic hypercalciuric stone-forming rats [21]. In a previous study, DNA sequence analysis of the coding region of* TRPV5* showed a novel T-to-C transition in codon 682 of* TRPV5*, mutating a conserved serine to a proline (S682P) [40]. In the same report, after comparing wild-type mice to heterozygous and homozygous mutant mice, there was evidence of hypercalciuria, polyuria, and a more-acidic urinary pH [40].

Limited clinical studies about the association between* TRPV5* genotypes and hypercalciuria are available. The first report investigated four* TRPV5* polymorphisms in Americans of African descent, where four nonsynonymous SNPs, A8V, R154H (rs4236480), A563T, and L712F, were investigated [19]. Only one variation was found to be significant: A563T of* TRPV5* inferred a higher calcium ion reabsorption ability among African Americans, giving people of African descent a conservative superiority for calcium influx over other ethnicities [19]. Another report with 20 French hypercalciuria patients found nonsignificant functional impacts of three* TRPV5 *SNPs (A8V, R154H, and A561T) [25] compared to wild-type normal* TRPV5* [25]. However, the limited set of human subjects' assigned may cause false-negative results. Our results suggest the role of gene polymorphism in increasing the presence of multiple stone numbers in patients carrying this nonsynonymous variation. The results may support a role of* TRPV5* in the development of calcium nephrolithiasis in Taiwanese population.

There were a few limitations to this study. First, we observed no association between the rs4236480 polymorphism and increased serum and/or urine levels of calcium in this cohort of Taiwanese patients. Recent genome wide studies have shown how tremendous cumulative factors contribute to serum/urine calcium concentration. O'Seaghdha et al. provided evidence that indicated several polymorphisms in vital genes for calcium homeostasis [41, 42]. Due to polygenic nature of calcium nephrolithiasis, it is difficult to detect a genetic association between one gene and urine/serum calcium concentration with a modest sample size. Additionally, the analysis for stone recurrence generated a null result. It might be, however, a false-negative result from our recurrence analysis due to the limitation of retrospective data collection. A conceivable justification for our current results was the difference in the genetic context of the cohort population and the possible polygenic complexity of nephrolithiasis disease.

## 5. Conclusions 

In conclusion, our results indicated a significant association between rs4236480 and kidney-stone multiplicity and showed that rs4236480 is a relevant susceptibility indicator for stone multiplicity in nephrolithiasis patients.

## Figures and Tables

**Table 1:** Basic characteristics of patients with nephrolithiasis.

Characteristics	Nephrolithiasis subjects
Number of subjects	365
Sex: male, no (%)	250 (68.5%)
Age (years)	54.3 ± 12.0
Urine data	
Uric acid (mg/dL)	40.5 ± 23.7
Calcium (mg/dL)	10.1 ± 7.4
Creatinine (mg/dL)	86.3 ± 56.6
Serum data	
Uric acid (mg/dL)	6.5 ± 1.7
Calcium (mg/dL)	8.9 ± 0.7
Creatinine (mg/dL)	1.2 ± 0.9

**Table 2:** Association analysis of *TRPV5* rs4236480 and kidney stone multiplicity.

SNP	Genotype	Multiplicity (%)		Genotype *p *	Dominant *p *	Recessive *p *	Log-additive *p *
		Multiple stone	Single stone				
rs4236480	TT	2 (2.8)	0 (0.0)	0.0372^∗^	0.0271^∗^	0.1125	0.0167^∗^
	CT	34 (47.9)	19 (30.2)				
	CC	35 (49.3)	44 (69.8)				

The *p* value was adjusted for sex and age.

^∗^The *p* value was statistically significant.

**Table 3:** Association analysis of *TRPV5* rs4236480 and kidney stone recurrence.

SNP	Genotype	Recurrence (%)		Genotype *p*	Dominant *p*	Recessive *p*	Log-additive *p*
		Yes	No				
rs4236480	TT	1 (1.9)	2 (2.9)	0.6502	0.5016	0.6284	0.6462
	CT	20 (37.7)	19 (27.9)				
	CC	32 (60.4)	47 (69.1)				

The *p* value was adjusted for sex and age.

**Table 4:** Association analysis of *TRPV5* rs4236480 and clinical biochemical data.

SNP	Genotype	Urine creatinine (UCr; mg/dL)	Urine calcium (UCa; mg/dL)	UCa/UCr	Urine uric acid (UUA; mg/dL)	UUA/UCr	Serum creatinine (mg/dL)	Serum calcium (mg/dL)	Serum uric acid (mg/dL)
	TT	93.44 ± 76.47	5.53 ± 2.93	0.10 ± 0.10	42.45 ± 28.59	0.52 ± 0.11	1.36 ± 0.87	8.77 ± 0.21	5.72 ± 1.30
rs4236480	CT	85.85 ± 50.35	10.77 ± 8.42	0.14 ± 0.11	44.35 ± 26.63	0.54 ± 0.16	1.14 ± 0.73	8.96 ± 0.47	6.66 ± 1.93
	CC	97.36 ± 64.87	10.74 ± 7.39	0.15 ± 0.10	43.45 ± 25.22	0.50 ± 0.16	1.20 ± 0.99	8.87 ± 0.73	6.47 ± 1.67
*p* value		0.3983	0.2521	0.5720	0.9555	0.1162	0.4514	0.5055	0.4945

*n*: number of patients for the statistical analysis.

The *p* value (genotype model) corresponding to the likelihood ratio test was obtained from a comparison with the null model and was adjusted for sex and age.

**Table 5:** Association analysis of *TRPV5* rs4236480 and subgroups of clinical biochemical data.

*TRPV5* rs4236480	Genotype model			Dominant model		Log-additive model
	CC	CT	TT	CC	CT+TT	CC-CT-TT
Serum uric acid (%)						
>7.2 mg/dL	132 (61.7)	77 (36.0)	5 (2.3)	132 (61.7)	82 (38.3)	214 (96.8)
≤7.2 mg/dL	4 (57.1)	2 (28.6)	1 (14.3)	4 (57.1)	3 (42.9)	7 (3.2)
*p* value	0.3763 (0.3440)			0.8092 (0.5879)		0.2954
OR (95% CI)	1.17 (0.21~6.52)			0.83 (0.18~3.80)		0.60 (0.17~2.14)
Serum calcium (%)						
>10.2 mg/dL	1 (33.3)	2 (66.7)	0 (0.0)	1 (33.3)	2 (66.7)	3 (1.4)
≤10.2 mg/dL	132 (61.7)	76 (35.5)	6 (2.8)	132 (61.7)	82 (38.3)	214 (98.6)
*p* value	0.5614			0.3529		0.4734
OR (95% CI)	3.47 (0.31~38.95)			3.22 (0.29~36.07)		2.11 (0.33~13.40)
Serum creatinine (%)						
>1.3 mg/dL	29 (56.9)	20 (39.2)	2 (3.9)	29 (56.9)	22 (43.1)	51 (22.2)
≤1.3 mg/dL	115 (64.2)	60 (33.5)	4 (2.2)	115 (64.2)	64 (35.8)	179 (77.8)
*p* value	0.8519			0.8389		0.7234
OR (95% CI)	1.32 (0.69~2.53)			1.36 (0.72~2.57)		1.35 (0.77~2.36)
UCa/UCr^1^ (%)						
≥0.2	33 (63.5)	17 (32.7)	2 (3.8)	33 (63.5)	19 (36.5)	52 (25.5)
<0.2	91 (59.9)	57 (37.5)	4 (2.6)	91 (59.9)	61 (40.1)	152 (74.5)
*p* value	0.8490			0.7703		0.9008
OR (95% CI)	0.82 (0.42~1.61)			0.86 (0.45~1.65)		0.92 (0.52~1.65)

The *p* value corresponding to the likelihood ratio test was obtained from a comparison with the null model and was adjusted for sex and age.

^1^UCa/UCr, urinary calcium-creatinine ratio.

OR: odds ratio; CI: confidence interval.
